# Maintenance of Genomic Stability in Mouse Embryonic Stem Cells: Relevance in Aging and Disease

**DOI:** 10.3390/ijms14022617

**Published:** 2013-01-28

**Authors:** Claudia Giachino, Luca Orlando, Valentina Turinetto

**Affiliations:** Department of Clinical and Biological Sciences, University of Turin, Regione Gonzole 10, 10043 Orbassano, Turin, Italy; E-Mails: luca.orlando@unito.it (L.O.); valentina.turinetto@unito.it (V.T.)

**Keywords:** embryonic stem cells, genotoxic stress, DNA repair, aging, tumor

## Abstract

Recent studies have shown that mouse embryonic stem cells (mESCs) rely on a distinctive genome caretaking network. In this review, we will discuss how mESCs functionally respond to DNA damage and describe several modifications in mESC DNA damage response, which accommodate dynamic cycling and preservation of genetic information. Subsequently, we will discuss how the transition from mESCs to adult stem/progenitor cells can be involved in the decline of tissue integrity and function in the elderly.

## 1. Introduction

Embryonic stem cells (ESCs) are dynamically cycling cells derived from the blastocyst inner cell mass, are pluripotent and have a virtually unlimited self-renewal potential [1].

Similarly to any other cell in the organism, ESCs must constantly contend with genotoxic stress arising from both exogenous environmental stimuli and endogenous chemical reactions, such as reactive oxygen species (ROS) generated by cellular metabolism [2].

ESCs are critical for embryo formation and, upon differentiation, they ensure the lifetime maintenance of any tissues. Any misrepair of DNA damage in ESCs can be transmitted to their differentiated daughter cells, thereby compromising tissue integrity and function. In order to prevent acquisition of mutations that would be transmitted to multiple cell lineages, ESCs exploit several modifications in DNA damage response, which accommodate dynamic cycling and preservation of genetic information [3,4].

An overview of the genome caretaking network of mESCs is the purpose of the first part of this review; in the second part, the possible pathophysiological consequences of misrepairing events are discussed.

## 2. Hypersensitivity to DNA Damage: The First Watchtower

**Figure 1:**
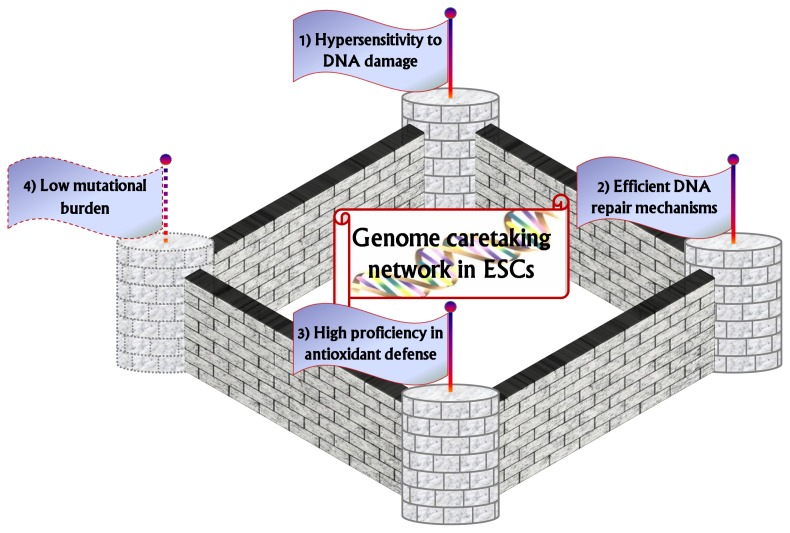
Optimal balance between maintaining sufficient numbers of embryonic stem cells (ESCs) (**2**–**4**) and eliminating severely damaged stem cells (**1**) as a major characteristic of the genome caretaking network in ESCs.

As a first mechanism contributing to preservation of genomic integrity, mESCs are hypersensitive to DNA damage and readily undergo either apoptosis or differentiation, thus removing damaged cells from the pluripotent pool (Figure 1 and Table 1). Mitotic tissues have evolved two major tumor suppressive mechanisms to limit the risk of malignant transformation: apoptosis and cellular senescence. Since they are immortal, ESCs cannot undergo replicative senescence. Whether they are capable of a telomere-independent senescence response remains to be elucidated, yet owing to the apparent lack of functional pRB protein in ESCs [5], the senescence pathway in general may be compromised. Instead, some intriguing observations indicate that ESCs have developed hypersensitivity towards apoptotic death and readily undergo apoptosis in response to the insults and/or inappropriate microenvironment, thus eliminating any cell at risk of malignant transformation. For example, treatment of mESCs with UV radiation or methylating agents results in the massive induction of apoptotic cell death [6–8]. Consistent with these observations, the known signaling pathways that mediate the G1 checkpoint are compromised in ESCs. After introduction of a genotoxic stress, such as a DNA double strand break, somatic cells activate two signaling pathways that lead to a G1 checkpoint arrest. A rapid response mechanism involves activation of ATM followed by activation of Chk2 kinase by ATM-mediated phosphorylation of threonine 68. The activated Chk2, in turn, phosphorylates Cdc25A phosphatase. In unperturbed cells, Cdc25A dephosphorylates Cdk2 at threonine 14 and tyrosine 15 to allow transit of cells from G1 into the S-phase. When Cdc25A is phosphorylated by Chk2 in stressed cells, it becomes ubiquitinylated and degraded in a proteosome-dependent manner, resulting in a G1 arrest [9]. A slower, but sustained response involves phosphorylation of p53 serine 15 by activated ATM and serine 20 by Chk2 and consequent induction of p21 transcription and G1 arrest. In contrast to somatic cells, both of these pathways are compromised in ESCs, partly as a consequence of Chk2 sequestration at centrosomes, which renders the Chk2 kinase unavailable to phosphorylate its substrates [10] and partly due to the sequestration of p53 in the cytoplasm, which impacts on the induction of p21 [4]. A possible consequence of the absence of a G1 arrest is that cells with DNA damage can transit from G1 into the S-phase, where the damage can be exacerbated by proceeding through a round of replication [6,11,12], thus causing apoptotic death. The exact mechanisms by which ESCs are poised to die following DNA damage, however, are not well understood. Studies have shown that pro-apoptotic protein BAX is maintained in an inactive form in the cytosol by several mechanisms, including the binding of NHEJ factor Ku70 [13,14]. Remarkably, in human ESCs (hESCs), Ku70 is constitutively acetylated, thus inhibiting its interaction with BAX. Moreover, constitutively active BAX is sequestered in the Golgi of hESCs, thus facilitating mitochondrial localization and rapid apoptotic signaling in the event of DNA damage [15].

Notably, the master apoptotic mediator p53 is only rarely involved ESC death. An emerging idea in the field of ESC biology, instead, is that p53 is a major driving force for the differentiation of ESCs, providing an alternative mechanism by which to eliminate damaged cells from the pluripotent stem cell pool. The connection between p53 and differentiation became particularly evident when Lin *et al*. found that p53 binds to the promoter of Nanog and suppresses its transcription in mESCs [16]. The homeodomain protein Nanog is highly abundant in ESCs and is required for self-renewal and maintenance of an undifferentiated state [32–34]. Suppression of Nanog transcription decreases the amount of Nanog protein and thus supports ESC differentiation. In addition to the Nanog promoter, p53 binds to the oct4 promoter, where it also reduces gene transcription [17]. Like Nanog, Oct4 belongs to the group of pluripotency factors that are necessary for maintaining ESCs in an undifferentiated state [32,35].

## 3. Efficient DNA Repair Mechanisms as the Second Watchtower

A second, complementary mechanism is the propensity of ESCs to have effectual processes for DNA repair, through upregulation of several DNA damage repair pathways (Figure 1 and Table 1).

### 3.1. Base Excision Repair

The base excision repair (BER) pathway repairs small base modifications, such as oxidative and alkylation damage and DNA single-strand breaks [36]. Genetic diseases caused by mutations in BER pathway genes are less common than those caused by other DNA repair pathway genes. Interestingly, however, 30% of all human tumors examined have variant POLβ, key proteins along the BER pathway, with approximately half having a single amino acid change [37]. An early link between an inherited defect in BER and cancer was reported in 2002, when a family with a phenotype similar to familial adenomatous polyposis was shown to have mutations in the gene encoding the human MutY homolog, a BER-associated adenine glycosylase active on G-A mispairs [38]. Repair is generally divided into two subtypes: short patch repair, where a single nucleotide is removed and replaced, and long patch repair in which a stretch of several nucleotides is removed from the damaged strand followed by DNA synthesis and ligation. The former is partially dependent upon DNA polymerase β to complete repair, while the latter is dependent upon PCNA and is independent of DNA polymerase β [39].

A limited number of studies have examined BER in ESCs, but it has been observed that basal levels of proteins involved in BER are significantly elevated in mESCs compared with mEFs [18]. Coupled with this elevation is a higher capacity of mESCs to repair an oligonucleotide template containing a uracil opposite a guanine *in vitro*, when compared to mEFs [18]. In another study, the repair kinetics of hESCs were significantly faster than human fibroblast cell lines (hEFs) when treated with a high dose of H2O2 and analyzed through the alkaline comet assay [19]. The mRNA levels for several BER genes were also significantly higher in untreated hESCs compared with hEFs [19]. However, when the rate of DNA incision *in vitro* by OGG1, a DNA glycosylase involved in BER, was measured using untreated extracellular extracts, no major differences in OGG1 activity were observed between hESCs and hEFs, and when hESCs and hEFs were treated with H_2_O_2_, the levels of both OGG1 and APE2 proteins were induced to barely higher levels after treatment in the hESCs [19].

### 3.2. Double Strand Break Repair

Double strand breaks (DSBs) in DNA are the most toxic type of DNA lesions a cell encounters [40]. Defects in the cellular response to DNA strand breaks underpin many human diseases, including disorders associated with cancer predisposition, immune dysfunction, radiosensitivity and neurodegeneration, two paradigmatic examples being Ataxia Telangiectasia (AT) and the Nijmegen Breakage Syndrome (NBS) [41].

DSB repair uses non-homologous end joining (NHEJ) or homologous recombination (HR) pathways [42–44]. HR uses a homologous DNA duplex, usually a sister chromatid, as the template for repair and is error-free. NHEJ repair joins ends directly in a process that is independent of extensive DNA sequence homology and is error-prone. It is not completely expected to have an error-prone pathway repair DSBs in ESCs. In keeping with this idea, a recent investigation showed that mESCs express lower levels of DNA-PKcs, compared to mEFs, which might contribute significantly in shifting the bias towards HR-dependent mechanisms [20]. Furthermore, ESCs lack a G1 checkpoint, have very short cell cycle G1 and G2 phases and spend about 75% of their cycle time in the S-phase [45]. The protracted proportion of time spent in the S-phase might also promote the preferential use of HR rather than NHEJ, since many of the proteins involved in HR also participate in DNA replication and are regulated by E2F. In addition, because of the very brief G1 phase, the majority of ESC genomes would have sister chromatids that would be available for efficient HR-mediated repair.

There are a number of studies that compare DSB repair mechanisms between ESCs and differentiated cells [46]. Initial DSB repair studies examined the steady state levels of different DSB repair proteins involved in HR and NHEJ pathways. Tichy *et al.* demonstrated that HR (RAD51, RAD52 and RAD54) and NHEJ (Ku70/Ku80) proteins are consistently elevated in mESCs, compared to mEFs, and when mESCs were functionally tested for the preferred pathway of DSB repair, they predominantly utilized the high fidelity homology-mediated repair pathway [21]. In contrast, expression of NHEJ protein DNA ligase IV is downregulated in mESCs compared to mEFs. Interestingly, when mESCs are differentiated in the presence of all-*trans* retinoic acid, DNA ligase IV expression levels increase, and NHEJ is reactivated and becomes the predominant pathway for repair of DSBs [21]. HR appears to be the predominant pathway choice to repair induced or spontaneous DNA damage throughout the mESC cycle in contrast to fibroblasts, where it is restricted to replicated chromatin [47]. This suggests that alternative templates, such as homologous chromosomes, are more frequently used to repair DSB in mESC. hESCs also have efficient DSB repair that is largely HR-mediated; yet hESCs rely on ATR, rather than ATM for regulating DSB repair, and this relationship dynamically changed as cells differentiated [22]. In addition, it was demonstrated that repair at a targeted DSB is highly precise in hESCs, compared to either the somatic human cells or murine embryonic stem cells, while differentiation of hESCs harboring the targeted reporter into astrocytes reduces both the efficiency and precision of repair [48].

Recently, it has been demonstrated how the histone H2AX phosphorylation (γH2AX), the earliest indicator of DNA DSBs, is expressed at a high basal level in mouse pluripotent stem cells. Interestingly, this basal γH2AX is not linked with the canonical DSB response pathway [49], but is important in the regulation of pluripotent stem cell self-renewal [50] and proliferation [51]. The mechanism behind the high basal γH2AX is not clear yet, and its implication in the embryonic stem cell genetic stability has to be further investigated.

### 3.3. Mismatch Repair

Mismatch repair (MMR) corrects base-base mismatches and insertion/deletion loops formed by misincorporation or strand slippage during DNA replication [52,53]. Mutations within genes in this repair pathway generally lead to hereditary nonpolyposis colorectal cancer (HNPCC) [54,55].

Analysis of mutation frequencies in cells proficient or deficient for components of the MMR pathway revealed a key role for MMR in the maintenance of mESC genomes. As an example, the frequency of spontaneous mutation increased from 10^−6^ in wild-type mESCs to 10^−4^ in mESCs lacking MSH2, a critical recognition component of the MMR pathway [56]. Many proteins involved in the MMR pathway were found to be expressed at high level under basal conditions in mESCs when compared with mEFs, as were several mRNA transcripts encoding these proteins [18]. When a plasmid-based fluorescent reporter containing a mismatch [57] was used to measure MMR activity in untreated mESCs or differentiated mEFs, the mESCs displayed about a 15-fold higher MMR activity over mEFs [18].

In another work, MMR has been compared after treatment with the methylating agent *N*-methyl-*N′*-nitro-*N*-nitrosoguanidine (MNNG) between undifferentiated ESCs, ESCs whose differentiation was induced with retinoic acid, 3T3 cells and mEFs [8]. A two-fold higher induction of apoptosis was evidenced in MNNG-treated ESCs compared with other cell types, and protein expression analysis revealed significantly elevated levels of the two MMR proteins Msh2 and Msh6 in ESCs compared with 3T3 cells. Interestingly, MNNG lesions are not repaired by MMR, thus suggesting that MMR protein enhancement is functional to the modulation of apoptosis after this type of damage. The possible link between expression of high levels of MMR proteins and apoptosis sensitization was confirmed through Msh2 overexpression in 3T3 cells and demonstration of their increased sensitivity to MNNG. It has been suggested that hyperphosphorylation of RB, resulting in more E2f1 binding to the Msh2 promoter, might be the mechanism by which the high level of Msh2 exerts its effect in ESCs. When ESCs were treated with MNNG after induction of differentiation, the cells had reduced levels of Msh2 and displayed a lower frequency of apoptosis compared to their undifferentiated counterparts [8], indicating that levels of Msh2 protein can direct cells to either repair their DNA or to undergo apoptosis. This example within the MMR pathway provides an unexpected link between the overexpression of repair proteins and the hypersensitivity to genotoxic stress typical of ESCs.

### 3.4. Nucleotide Excision Repair

The nucleotide excision repair (NER) pathway repairs bulky, helix-distorting DNA lesions, particularly DNA damage resulting from UV radiation damage [58,59]. Defects in NER result in the human disorder Xeroderma Pigmentosum, Cockayne’s Syndrome and Tricothiodystrophy [41].

When tested for their capacities to repair damage induced by UV light, mESCs and mEFs behave differently. Many years ago, Pedersen and Cleaver demonstrated that cells of the blastocyst inner cell mass, from which ESCs are isolated, underwent only minimal unscheduled DNA synthesis following UV irradiation compared with other cells of the blastocyst [60]. It was shown later that when ESCs are exposed to high dose UVC, severely damaged cells are rapidly eliminated by apoptosis [7]. It is unclear at the moment if the hypersensitivity of mESCs to UV and subsequent cell death is or not the result of defective DNA repair. Indeed, transcription-coupled repair, a subpathway of NER, is functional in wild-type mESC, as demonstrated using mutation frequency assays at the Hprt locus in wild-type mESCs or mESCs that were deficient for components of the NER machinery [23]. Results indicated that mutant mESCs treated with different dosages of UV displayed mutation frequencies that ranged from one- to four-fold higher than wild-type cells processed in the same manner. On the other hand, Van Sloun *et al*. showed that repair of cyclobutane pyrimidine dimers (CPD) in transcribed genes could not be detected in mESCs, whereas the removal of (6-4) photoproducts (6-4PP) was incomplete, already reaching its maximum (30%) 4 h after irradiation. Measurements of repair replication revealed a saturation of NER activity at UV doses >5 J/m^2^, while at lower doses, the repair kinetics were similar to those in mEFs. Possibly, to avoid the accumulation of mutated cells, ESCs rely on the induction of a strong apoptotic response with a simultaneous shutting down of NER activity [7].

## 4. The Third Watchtower: High Proficiency in Antioxidant Defense

Endogenous factors associated with cell division and metabolism are attributed to the major sources of DNA damage in stem cells. Among the most significant endogenous mutagens are reactive oxygen species (ROS), including superoxide anions, hydrogen peroxide, organic peroxides and hydroxyl radicals. Normal metabolic processes, such as oxidative phosphorylation and nucleotide catabolism, are responsible for continuous ROS generation in these cells. An additional mechanism of ROS production relies on specific plasma membrane oxidases acting in response to growth factors or cytokines. If not inactivated in a timely manner by cellular anti-oxidative systems, ROS may cause DNA damage.

Numerous evidences suggests that ESCs possess very efficient antioxidant defense pathways (Figure 1 and Table 1). mESCs were shown to be capable of withstanding supraphysiological levels of oxygen, an ability that rapidly decreased during the early steps of differentiation into embryoid bodies [24]. Consistently, intracellular ROS levels were shown to increase after differentiation, and this was correlated to decreased mRNA levels of ROS inactivating enzymes, as emerged from a search for gene expression changes associated with ESC differentiation [24]. These data were corroborated by independent works detecting an increase in ROS levels in various models of ESC differentiation [61,62]. In addition, when mESCs were cultured under hyperoxic conditions (40% oxygen), they were shown capable of continued proliferation, while mEFs underwent senescence under the same conditions [24].

An interesting biphasic relationship between the net intracellular levels of ROS and genomic stability in ESCs has been described recently. It was found that mild ROS suppression obtained by culturing ESCs in physiological oxygen content (5%) decreased karyotypic abnormalities if compared to 20% oxygen cultures, while profound ROS suppression obtained by supplementation with antioxidants paradoxically enhanced genomic alterations [63]. Oxidative stress is well-known to induce DNA damage, accounting for the high frequency of karyotypic abnormalities observed when ESCs are cultured in 20% O_2_ culture. On the other hand, excessive suppression of ROS to sub-physiological levels downregulates DNA repair pathways, thereby contributing to genomic instability. These results suggest a new concept that optimal “physiological” levels of ROS are required for activation of DNA repair pathways and maintenance of genomic stability in stem cells [63].

## 5. Low Mutational Burden as a Putative Fourth Watchtower?

Suppression of mutagenesis in mESCs might represent another mechanism that contributes to preservation of genomic integrity (Figure 1 and Table 1). Through the use of a selection-based assay and employing isogenic mouse embryo fibroblasts (mEFs) as comparison, Cervantes *et al.* [64] demonstrated that mESCs display about 100-fold lower spontaneous mutation frequency at the Aprt reporter locus (from 10^−4^ in mEFs to 10^−6^ in mESCs). Most of the observed mutational events involved loss of heterozygosity (LOH) due to mitotic recombination, with point mutations and deletions making up the remainder. When spontaneous mutation frequencies at the Hprt gene was assessed through a similar approach, spontaneous mutation in ESCs was undetectable (below 10^−8^), whereas mutation frequency in mEFs was in the range of 10^−5^. In this case, there was no contribution of LOH to the observed mutation frequencies, as Hprt is located on the X chromosome and the cells used in this study were derived from male embryos. This difference probably accounts for much of the discrepancies observed in mutation frequency between the two loci, Aprt and Hprt. Other reports, however, do not support the evidence that mESCs display lower mutation frequencies. For example, mutation frequencies at the Rosa26 locus turned out to be similar between mESCs and mEFs (about 10^−4^) using fluorescent protein reporter-based technologies [65,66]. It is unclear at the moment whether these contrasting findings are unique at the Rosa26 locus or can be attributed to the different approaches used to quantify mutation frequencies. Differences in the ESC lines used could also account for these discrepancies. In addition, when the ability of carcinogenic agents to induce loss-of-heterozygosity (LOH) in diploid mESCs was examined, Donahue *et al*. found that brief exposures to nontoxic levels of several carcinogens stimulated genome-wide LOH, with maximum per-gene frequencies approaching one percent [67]. Due to these divergent opinions, it is not yet possible to conclude about the role of suppression of mutagenesis as a further mechanism protecting ESCs from genomic instability; future genome-wide studies will be needed to clarify this still debated point.

## 6. Relevance in Aging and Disease

**Figure 2:**
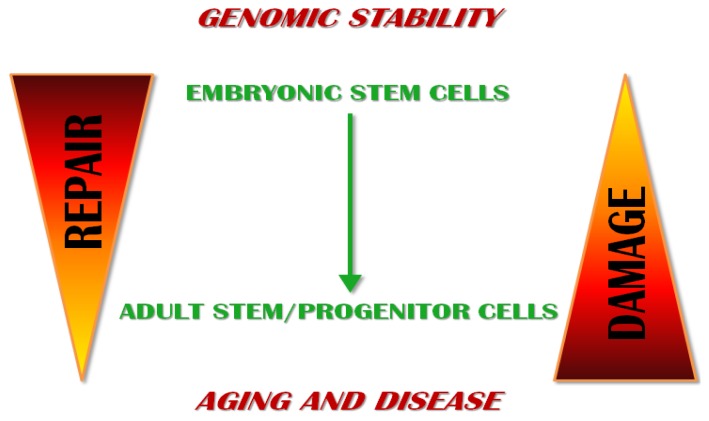
Hierarchy of cell differentiation and DNA damage/DNA repair functions. ESCs show less inherent DNA damage and a higher DNA repair capacity compared to adult stem/progenitor cells. DNA misrepair activity in adult stem/progenitor cells may lead to aging and diseases (tumors, degenerative diseases).

ESCs are critical for embryo formation and, upon differentiation, they ensure the lifetime maintenance of any tissues. Any misrepair of DNA damage can be transmitted to their differentiated daughter cells, thereby compromising tissue integrity and function. Mutations that diminish the renewal and/or differentiation potential of ESCs might result in tissue atrophy and aging phenotypes, whereas mutations providing a selective advantage to the mutated cells can lead to cancer development (Figure 2 and Table 1).

### 6.1. Aging and ESCs

Aging is a complex biological process resulting from gradual changes in the phenotype and functions of cells. The age-associated pathophysiological changes usually lead to an imbalance that favors the mechanisms of replicative senescence and apoptosis rather than DNA repair mechanisms (reviewed in [68]). However, while senescence is invariable in most cells, including transformed cells [69], ESCs do not have demonstrable senescence [70]. Several pathways that are activated independently or together with others can allow the cells to bypass senescence: the telomerase pathway required to maintain telomere ends [71], the p53 and Rb pathways needed to direct senescence in response to DNA damage [72], telomere shortening and mitotic signals [71] and the insulin-like growth factor-Akt pathway that may regulate lifespan, cell proliferation and the mitochondrial/oxidative stress pathway [73,74]. By contrast with every other cell types, ESCs express inactive p53 and Rb, active Akt and maintain telomerase and telomere length.

Thus, it is the aging of adult stem/progenitor cells that is actually believed to be central to the decline of tissue integrity and function in the elderly [31].

### 6.2. Aging and Adult Stem/Progenitor Cells

As described, ESCs exhibit an unlimited self-renewal capacity and a substantial resistance to genomic instability and malignant transformation. During early development, ESCs differentiate into cells that form all the tissues of the future organism, including adult stem/progenitor cells. In this process, cells lose their pluripotent potential and unlimited proliferating capacity. Transition from ESCs to adult stem/progenitor cells may have involved the evolutionary trade-off: senescence prevents cancer, but may promote aging [75].

Genetic alterations accumulated in adult stem/progenitor cells during chronological aging may result in their loss, or acquisition of a dysfunctional behavior [76–79], including triggering of different cell cycle checkpoint mechanisms [77,79], among these, an upregulation of tumor suppressor gene products, like p16INK4A and p19ARF, two alternatively spliced proteins encoded by the CDKN2A/ARF locus on human chromosome 9p21 and p53. The activation of their corresponding signaling pathways may result in cell growth inhibition or senescence through different mechanisms, such as an inhibition of activities of cyclin-dependent kinase (CDK)/cyclin complexes and repression of the free E2F transcription factor-induced gene products [25–28]. Elevated expression of the Rb effector p16INK4A has been found in numerous tissues with age [29,30] and has emerged as one of the most important aging biomarkers, along with senescence-associated β-galactosidase activity (SA-b-gal) [80]. According to several studies, p16INK4A increases in many stem cell compartments with age, and this induction has functional consequences. Neural stem cells in the subventricular zone of the mammalian brain diminish in number and function with age [27,81]. In another study, p16INK4A expression was elevated in hematopoietic stem cells from old mice, whereas age-associated repopulating deficits were improved in aged p16INK4A-deficient mice [82]. The question of how p53 impacts stem cell aging is more complex. Loss of p53 surely predisposes to different forms of neoplasms [83], whereas mice overexpressing a truncated activated form of p53 [84] exhibit suppression of tumorigenesis, yet develop early degenerative phenotypes reminiscent of aging. On the other hand, mice with increased, but normally regulated, expression of p53 are resistant to tumorigenesis, live longer and have reduced levels of age-associated damage to proteins, lipids and DNA [85]. Mice with increased p53 activity resulting from a hypomorphic allele of Mdm2 are also tumor resistant, yet age normally [86]. Thus, modulation of p53 activity can have either proaging or antiaging effects depending on context [31]. In accordance with these results, adult stem/progenitor cells in mice expressing a truncated p53 protein with elevated activity showed reduced proliferative and repopulating capacity compared to wild-type controls, while the same cells from p53^+/−^ mice exhibited increased activity [87]. In addition, activation of the p53 transcription factor may trigger the apoptotic death program in adult stem/progenitor cells and their progenies. The mechanisms are an upregulation of diverse pro-apoptotic factors, such as Bax and Bak proteins, downregulation of anti-apoptotic proteins, like Bcl-2 and Bcl-xL, or an enhanced expression of the p53 upregulated modulator of apoptosis (PUMA) and Noxa [88,89]. Interestingly, an activation of the tumor suppressing pathways cited above in response to oncogenic events, like activating mutations in Ras and Myc oncoproteins, may also help prevent cancer initiation [26,88,90–92].

### 6.3. Disease and ESCs

Cancer is thought to originate in stem cells through the accumulation of multiple mutations, some of which result in a loss of heterozygosity (LOH). It has been demonstrated that exposure of mESCs to nontoxic amounts of mutagens triggers a marked increase in the frequency of LOH [67]. The authors used a mESC line to establish a panel of p53 clones, each containing a neomycin-resistance cassette inserted at a different chromosomal locus. Brief exposure of the clones to a variety of carcinogens resulted in LOH at high frequencies (1 in 8000 cells), acquired through different mechanisms, such as deletions, recombination, chromosomal rearrangements or even point mutations [93]. In the mESCs analyzed, LOH was scored only if it resulted in duplication of the neomycin marker, and the results suggest that noninherited cancers could arise from prior exposure to genotoxic agents and that the high incidence of LOH might obviate the requirement for a mutator phenotype. Alternatively, the high incidence of LOH might result from an experimentally-induced mutator phenotype, though there is consistent evidence against this possibility: the frequency of LOH at a second reporter gene, thymidine kinase, was not increased in cells that had previously undergone spontaneous or carcinogen-induced LOH. Thus, mutagen induction of LOH in mESCs suggests a new pathway to account for the multiple homozygous mutations in human tumors [94]. The use on mESCs in studies of carcinogen-induced LOH is potentially important for several reasons [95]. First, increasing evidence suggests ESCs, with an intrinsic capacity for self-renewal and their immediate progeny, constitute the principal targets for oncogenic mutations, and stepwise deregulations of stem cell functions is thought to underlie the onset and progression of neoplasia [96]. Second, as discussed in the first part of this review, ESCs possess specialized mechanisms to suppress mutations, possibly as a defense against oncogenic transformation [97]. Template maintenance, a mechanism mediated by asymmetric chromosome segregation through which stem cells in several tissues appear to maintain legacy DNA templates even while transmitting newly synthesized DNA sequences to their daughter cells, has been described in ESCs [98]. However, other features of ESCs could have the opposite effect. Chromosome segregation requires topoisomerases to untangle concatenated DNA sequences during mitosis, and in the presence of topoisomerase inhibitors, many cell types arrest in the G2 phase of the cell cycle, a response attributed to a DNA concatenation checkpoint. Remarkably, ESCs lack this checkpoint [99], creating a potentially serious source of chromosome instability.

### 6.4. Disease and Adult Stem/Progenitor Cells

DNA damage and its consequences in ESCs do not affect fitness immediately, since stem cells are not involved in carrying out functions specific for target tissues. More likely, mutations or epimutations accumulated in the ESCs as a result of genome maintenance errors are transmitted to daughter cells that become the newly differentiated cells, and this impacts tissue functionality by adversely affecting the transcriptome. The increasing load of mutations/epimutations in the proliferation-capable ESCs increases the risk of neoplastic transformation, the price for extended longevity.

Evidence from a number of experiments has revealed that the occurrence of genetic alterations in adult stem/progenitor cells may result in their malignant transformation into tumorigenic cells also designated as cancer-initiating cells [100,101]. The identification of small subpopulations of immature cells with stem cell-like properties from fresh patients’ tumor tissues and established cancer cell lines, comprising about 0.1%–3% of total cancer cell mass, supported the critical functions of cancer-initiating cells in cancer development [102–105]. Cancer-initiating cells typically expressed specific stem cell-like markers, such as CD133, CD44 and stem cell factor (SCF) receptor KIT, were telomerase-positive and lacked differentiation marker expression. It has also been shown that cancer-initiating cells were able to give rise to the complete mass of differentiated cancer cells *in vitro* and recapitulated the morphological and phenotypic characteristics of the original tumors in animal models *in vivo* [104,106–110]. As an additional proof of concept, prolonged *in vitro* culture of adult stem/progenitor cells promoted the appearance of a transformed phenotype. Miura *et al*. obtained cancer progenitor cells from bone marrow derived mesenchymal stem cells after long-term culture [111], and these cancer progenitor cells formed fibrosarcoma *in vivo*. The mechanism of transformation was associated with accumulated chromosomal abnormalities and increased c-Myc expression [111], suggesting an association between cancer progenitor cells and genomic instability. A similar association was also observed after a long-term culture of human adult non-tumorigenic neural stem cells by Shiras *et al.* The concurrent emergence of a high level of genomic instability and a spontaneously immortalized clone was observed, which developed into a cell line with features of cancer stem cells, including the capacity to form CD133 positive neurospheres and development into intracranial tumors [112].

Progression of cancer is normally accompanied by an accumulation of additional transforming mutations in cancer-initiating cells and their progenies concomitant with the changes in their microenvironment conferring to them a more aggressive phenotype [110,113–115]. Within this matter, the long lifespan of adult stem/progenitor cells may allow them to accumulate distinct genetic alterations that are necessary for their malignant transformation in cancer-initiating cells and development of certain age-related cancer types.

Altogether, these data indicate that age-related loss of stem cells, age-related stem cell dysfunction and age-related neoplastic transformation of stem cells could all be adverse consequences of the adult stem/progenitor cell response to DNA damage.

## 7. Conclusions

ESCs need to maintain genomic integrity so that they can retain the ability to differentiate into multiple cell types without propagating DNA errors. Several lines of evidence suggest that the genome caretaking network of mESCs is a peculiar one.

Different strategies are concurrently employed to maintain a stable genome and to prevent passing of genomic aberrations on to the progeny. Loss of damaged self-renewing cells due to hypersensitivity to DNA damage appears to be the first strategy that effectively maintains the proliferating cell population genetically pristine. The absence of a G1/S cell cycle arrest promotes the apoptotic response of damaged cells before DNA changes can be fixed in the form of mutation during the S-phase, while p53-mediated differentiation provides an alternative mechanism of elimination of damaged cells from the pluripotent stem cell pool. In addition, several studies have suggested that some DNA repair pathways and antioxidant defense are superior in ESCs compared with various differentiated cells. ESCs express a higher level of DNA repair proteins and exhibit enhanced repair of multiple types of DNA damage; on the other hand, an efficient oxidative damage sensor system directs the ESC fate from repair to apoptosis depending on the levels of intracellular ROS. Finally, suppression of mutagenesis in ESCs might represent another putative strategy of genome surveillance, as distinct differences in mutation frequencies between ESCs and somatic cells have been reported for some loci; however, due to contrasting literature findings, this last issue awaits confirmation.

While ESCs rely on several modifications in DNA damage response to accommodate dynamic cycling and preservation of genetic information, the adult stem/progenitor cell compartment that emerges after their differentiation may not. Mutations that diminish the renewal and/or differentiation potential of adult stem/progenitor cells can result in tissue atrophy and aging phenotypes, whereas mutations providing a selective advantage to the mutated cells can lead to cancer development.

## Figures and Tables

**Table 1:** Main signaling pathways involved in stem cell maintenance of genomic stability, in aging and disease.

ESCs hypersensitivity to DNA damage			

Mechanism	Pathway involved	Species	References
G1 arrest impairment	Chk2 centrosome sequestration; p53 cytoplasm sequestration.	Mouse	[4,10]
Rapid apoptotic response following DNA damage	Constitutively activated form of BAX	Mouse, Human	[13–15]
Differentiation	p53 mediated repression of Nanog and Oct4 promoter	Mouse, Human	[16,17]

**ESCs DNA repair mechanisms**			

**Mechanism**	**Pathway involved**	**Species**	**References**

High efficiency in Base Excision Repair	BER pathway proteins over expressed in ESCs	Mouse, Human	[18,19]
Preferential repair of DSBs through HR rather than NHEJ	High level of HR pathway proteins, such as RAD51, RAD52, RAD 54	Mouse	[20,21]
	ATR dependent HR	Human	[22]
High efficiency in Mismatch Repair	High basal level of Msh2 and Msh6	Mouse	[18]
Fine regulation of Nucleotide Excision Repair	Shutting down of NER activity when high amount of DNA damage occurs	Mouse	[7,23]

**ESCs high proficiency in antioxidant defense**			

**Mechanism**	**Pathway involved**	**Species**	**References**

Ability to proliferate in hyperoxic conditions	High level of ROS inactivating enzymes	Mouse	[24]

**Adult/progenitor stem cells aging and disease**			

**Mechanism**	**Pathway involved**	**Species**	**References**

Age associated pathophysiological changes	Upregulation of tumor suppressor gene products like p16INK4A and p19ARF Misregulation of p53 levels	Mouse, Human Mouse, Human	[25–30] [31]
